# Service Development and Implementation: Learning From Diverse Case Examples

**DOI:** 10.1093/geroni/igaa057.2496

**Published:** 2020-12-16

**Authors:** Liat Ayalon, Mary Wyman, Anne Martin-Matthews

## Abstract

The development of health and social services should be based on a thorough needs assessment with all stakeholders, followed by ongoing monitoring of implementation and subsequent short and long term outcomes. Relying on four different service models, this symposium reviews their evaluation processes and summarizes the main lessons learned, in order to inform future efforts. Wyman and colleagues outline efforts to develop culturally-sensitive dementia care services for American Indian and Alaska Native older adults and caregivers using qualitative interviews and community-based participatory research methods. Findings offer guidance for culturally-tailored implementation of services. Shepherd-Banigan et al. report on a quantitative needs assessment of over 1,500 caregivers of older Veterans enrolled in Veterans Administration healthcare. Providing care to individuals with co-morbid conditions predicted higher levels of distress and burden among caregivers, pointing to likely benefit from additional caregiver-focused support programs. The paper by Gum and co-authors moves to assessment of outcomes by examining mortality and other outcomes among older adults screened by an Area Agency on Aging and either receiving or waiting for services. Finally, the paper by Ayalon and Shinan-Altman use service evaluation to demonstrate the importance of needs assessment and the gap between the vision of service developers and real life constraints. The included papers discuss the value of various methodologies, illustrating the important role that assessment and evaluation play in service development and implementation for older adults and caregivers.

